# Vascular endothelial growth factor (VEGF) expression in locally advanced prostate cancer: secondary analysis of radiation therapy oncology group (RTOG) 8610

**DOI:** 10.1186/1748-717X-8-100

**Published:** 2013-04-25

**Authors:** Larry Pan, Seunghee Baek, Pamela R Edmonds, Mack Roach, Harvey Wolkov, Satish Shah, Alan Pollack, M Elizabeth Hammond, Adam P Dicker

**Affiliations:** 1 Department of Radiation Oncology, Prince Edward Island Cancer Treatment Centre, Charlottetown, PEI; and Dalhousie University, Halifax, NS, Canada; 2 Department of Biostatistics, University of Pennsylvania, Philadelphia, PA, USA; 3 Pathology, Abington Hospital, Abington, PA, USA; 4 Radiation Oncology, University of California, San Francisco, San Francisco, CA, USA; 5 Radiation Oncology, Sacramento, CA, USA; 6 Radiation Oncology, Merle M. Mahr Cancer Center, Madisonville, KY, USA; 7 Radiation Oncology, University of Miami, Miami, FL, USA; 8 Intermountain Medical Center, Salt Lake City, UT, USA; 9 Radiation Oncology, Thomas Jefferson University Hospital, Philadelphia, PA, USA

**Keywords:** Prostate cancer, Vascular endothelial growth factor, Prognostic biomarker, Predictive biomarker, Radiation therapy

## Abstract

**Background:**

Angiogenesis is a key element in solid-tumor growth, invasion, and metastasis. VEGF is among the most potent angiogenic factor thus far detected. The aim of the present study is to explore the potential of VEGF (also known as VEGF-A) as a prognostic and predictive biomarker among men with locally advanced prostate cancer.

**Methods:**

The analysis was performed using patients enrolled on RTOG 8610, a phase III randomized control trial of radiation therapy alone (Arm 1) versus short-term neoadjuvant and concurrent androgen deprivation and radiation therapy (Arm 2) in men with locally advanced prostate carcinoma. Tissue samples were obtained from the RTOG tissue repository. Hematoxylin and eosin slides were reviewed, and paraffin blocks were immunohistochemically stained for VEGF expression and graded by Intensity score (0–3). Cox or Fine and Gray’s proportional hazards models were used.

**Results:**

Sufficient pathologic material was available from 103 (23%) of the 456 analyzable patients enrolled in the RTOG 8610 study. There were no statistically significant differences in the pre-treatment characteristics between the patient groups with and without VEGF intensity data. Median follow-up for all surviving patients with VEGF intensity data is 12.2 years. Univariate and multivariate analyses demonstrated no statistically significant correlation between the intensity of VEGF expression and overall survival, distant metastasis, local progression, disease-free survival, or biochemical failure. VEGF expression was also not statistically significantly associated with any of the endpoints when analyzed by treatment arm.

**Conclusions:**

This study revealed no statistically significant prognostic or predictive value of VEGF expression for locally advanced prostate cancer. This analysis is among one of the largest sample bases with long-term follow-up in a well-characterized patient population. There is an urgent need to establish multidisciplinary initiatives for coordinating further research in the area of human prostate cancer biomarkers.

## Introduction

Prostate cancer represents the most common malignancy and the second leading cause of cancer-related death in American men over the age of 40 years [1]. The majority of deaths secondary to prostate cancer are from metastases as a result of lymphatic or hematogenous dissemination of tumor cells. Angiogenesis, the development of new vessels from existing vasculature, is a key element in solid-tumor growth, invasion, and metastasis [2,3]. A balance of proangiogenic and antiangiogenic factors tightly controls physiologic angiogenesis to achieve homeostasis. A tipping of this balance towards pro-angiogenesis, termed the “angiogenic switch”, occurs with inflammation, tissue hypoxia, or neoplasia [3,4].

Vascular endothelial growth factor (VEGF) is among the most potent angiogenic factor thus far detected and has been found to be highly specific for endothelial cells in vitro and in vivo, promoting endothelial cell proliferation and increasing vascular permeability [5-9]. VEGF is a 45 kDa heparin-binding polypeptide of the platelet-derived growth factor family and is secreted by a variety of malignant cells. It has been shown to be expressed in many different types of tumors, including renal cell carcinoma, breast carcinoma, gliomas, and hepatocellular carcinoma [10-13].

By diffusion of oxygen and metabolites from local vessels, tumor cells can survive only up to a tumor diameter of 2–3 mm, beyond which new vessel formation is required [14]. As a result of the ensuing hypoxia, hypoxia inducible factor 1-alpha (HIF-1a) stabilizes and translocates to the nucleus to bind with HIF-1b forming HIF. This latter transcription factor then stimulates VEGF production, resulting in angiogenesis and the development of new vessels into the tumor [3,4,15,16]. Recently, it has also been suggested that androgen deprivation therapy may result in increased VEGF expression, suggesting the potential utility of VEGF-based anti-angiogenic agents together with androgen deprivation in the management of advanced prostate cancer [17]. The VEGF expression of a tumor may thus potentially have prognostic and predictive value for patient outcomes and tumor specific therapies.

Although VEGF expression in prostate adenocarcinoma has been shown to be expressed by immunohistochemical (IHC) analysis in various studies, the results in the literature have been markedly variable, with prior studies reporting VEGF expression in approximately 40% to 100% of prostate cancer cases [18-28]. The aim of the present study is to explore the potential value of VEGF as a prognostic and predictive biomarker among men with locally advanced prostate cancer enrolled on a phase III trial in the Radiation Therapy Oncology Group (RTOG), RTOG 8610.

## Methods

### Study population

The analysis was performed using patients enrolled on RTOG 8610 [29] (“a phase III trial of goserelin and flutamide used as cytoreductive agents in locally advanced carcinoma of the prostate treated with definitive radiotherapy”), which closed in 1991 with a total of 471 patients entered; 456 of the patients were assessable. Patients received short-term androgen deprivation therapy (STAD) consisting of 4 months of hormonal therapy given neoadjuvantly and concurrently with radiation therapy (RT), or they received RT alone. Results from the trial demonstrated a statistically significant improvement in 10-year biochemical failure (65% vs. 80%; p < 0.0001), disease-free survival (11% vs. 3%; p < 0.0001), disease-specific mortality (23% vs. 36%; p = 0.01), and distant metastasis (35% vs. 47%; p = 0.006) with the addition of STAD. Diagnostic material (from needle biopsies or transurethral resections) was reviewed centrally for 461 (98%) of the 471 patients by the study pathologist (D. J. Grignon, Wayne State University), and the tumors were graded according to the criteria of Gleason [30]. Tissue blocks were requested from participating institutions (>100) at the time of central pathology review for all cases that were reviewed. For the present retrospective study, a subset of the patients with sufficient pathologic material available entered in the RTOG 8610 protocol was evaluated for VEGF expression (103 patients). Specimens on all available individual patients enrolled on the RTOG 8610 protocol were obtained from the RTOG tissue repository.

### Immunohistochemical (IHC) analysis

Formalin-fixed, paraffin-embedded tissue from the pre-treatment diagnostic biopsies was sectioned and stored at 4°C, for more than 5 years in the majority of cases, before being processed for IHC staining. The paraffin blocks were used to prepare the Hematoxylin and Eosin stained slides. These slides were reviewed by one of the investigators (E.H.) to select regions of invasive tumor without inflammation or necrosis. Regions with such tumor areas were outlined with a cytology marking pen. Subsequent sections for use in immunohistochemistry were cut at 4 microns. For inclusion in the study, the stained section had to contain identifiable carcinoma. Positive control tissues for each reaction were also cut onto the test slide (kidney tissue), so that positive control and slide to be tested were simultaneously stained. Unstained sections were used in a standard IHC assay for VEGF involving heat induced epitope retrieval for 20 minutes, followed by antibody incubation with goat polyclonal VEGF antibody (RD systems, catalogue #AF-293-NA) at 1:20 for 30 minutes followed by labeled streptavidin biotin (LSAB) detection using Diaminobenzidine as substrate. The IHC procedure was done on a DAKO autostainer (DAKO, Inc, Carpenteria, CA).

Three investigators (E.H., P.E. and A.D.) reviewed all slides and recorded results without knowledge of patient outcome. Consensus was achieved on the scoring. Scale of 0-3+ was used to grade intensity of cytoplasmic staining relative to the staining of the control kidney tissue. Dark cytoplasmic staining was considered 3+. Weak staining was pale staining relative to kidney control and 2+ staining was any intensity between weak and strong staining. This is a conventional scale for intensity measurement used for most antigens. For statistical analysis, grades 0 and 1 were considered as negative, whereas grades 2 and 3 as positive.

### Definition of outcomes

The endpoints used in the analysis were per the RTOG 8610 protocol: overall survival (OS), local progression (LP), distant metastasis (DM), biochemical failure (BF), and disease-free survival (DFS). The failure event for overall survival was defined as death due to any cause. The overall survival time is measured from the date of randomization to the date of death or the date of last follow-up (censored). A local progression event is defined as an increase of more than 50% in tumor size (cross-sectional area), recurrence of a palpable tumor after initial clearance, or biopsy specimen revealing adenocarcinoma of the prostate two years or more after study entry. A distant metastasis event is defined as the clinical or radiographic evidence of disease beyond the pelvis while regional metastasis is a clinical or radiographic evidence of tumor in the pelvis with or without palpable tumor in prostate by digital rectal examination. Time to a distant and a local progression is measured from the date of randomization to the occurrence of either event or to the date of the most recent follow-up. Biochemical failure is defined as a PSA of > 1.5 ng/ml one year post-randomization. Time to biochemical failure is from the one year post-randomization date to a failure date. A failure in disease-free survival is defined as death due to any cause, local progression, biochemical failure, regional metastasis, or distant metastasis. Time to a disease-free survival is measured from the date of randomization to the earliest occurrence of all failure events or the most recent follow-up.

### Statistical analysis

VEGF expression intensity score data were dichotomized as follows: Negative (VEGF = 0–1) vs. Positive (VEGF = 2–3). The following covariates were considered in the multivariate analyses (MVA’s): age (<71 vs. ≥71), assigned treatment (RT vs. STAD + RT), Gleason Score (2–6 vs. 7–10), and clinical T-stage (T2 vs. T3). The Cox proportional hazard models were used for OS and DFS, or Fine and Gray’s proportional hazards models were used for LP, BF, and DM to examine if VEGF expression is associated with patient outcomes with and without covariates. These analyses were also performed by treatment group to see the predictive value of VEGF. The pre-treatment characteristics and outcomes were compared between the patient groups with and without missing VEGF values and between the VEGF negative and positive groups by Chi-square test statistics. Unadjusted and adjusted hazard ratios (HRs) were calculated for all covariates using either the Cox proportional hazard model or Fine and Gray’s regression model with 95% confidence intervals and p-values. All statistical tests were done at significance level of 0.05. R software was used for Fine and Gray’s model whereas Statistical Analysis System (SAS Institute, Car, NC) was used for the rest of the analyses.

## Results

From the RTOG tissue repository, tissue samples were obtained from 103 (23%) of the 456 analyzable patients enrolled in the RTOG 8610 study. All had pre-treatment characteristics available for analysis. The majority of patients had a clinical tumor stage T3 (75% of patients) and T2 for the remaining 25% of patients. Seventy-five percent of patients had Gleason Score (GS) 7–10, 24% had GS 2–6, and 1% unknown/missing. Fifty-three percent of patients were 71 years old or greater. There were no statistically significant differences in the pre-treatment characteristics between the patient groups with and without VEGF intensity data (not shown). Median follow-up for all surviving patients with VEGF intensity data is 12.2 years. A negative VEGF intensity score (0–1) was found in 47% of patients while a positive VEGF intensity score (2–3) was found in 53%. The distribution of VEGF intensity score was well balanced between treatment arms. In the RT alone arm, 45% and 55% of patients had a VEGF intensity score of 0–1 and 2–3, respectively, while in the STAD + RT arm, 49% and 51% had a VEGF intensity score of 0–1 and 2–3, respectively.

Table 1 illustrates the pre-treatment characteristics of patients who are eligible and with VEGF intensity score. There are no statistically significant differences between the VEGF negative and positive groups with respect to the pre-treatment characteristics.

**Table 1:** Pre-treatment characteristics by VEGF intensity score (n = 103)

	**Negative VEGF intensity (0–1)**		**Positive VEGF intensity (2–3)**		
	**(n = 48)**		**(n = 55)**		
**Characteristics**	**n**	**%**	**n**	**%**	p-value^*^
Age					
Median	71.5		71		
Range	55 – 77		55 – 81		
<71	21	44	27	49	0.588*
≥71	27	56	28	51	
Assigned Treatment					
STAD + RT arm	22	46	23	42	0.682
RT alone arm	26	54	32	58	
Combined Gleason Score					
Missing	0	0	1	2	
2-6	14	29	11	20	0.303**
7-10	34	71	43	78	
Clinical Stage					
T2	14	29	12	22	0.392*
T3	34	71	43	78	
Failures					
Overall Survival	39	81	51	93	
Distant Metastasis	25	52	31	56	
Local Progression	21	44	26	47	
Disease-free Survival	48	100	54	98	
Biochemical Failure	37	77	40	73	

*p-value is from Chi-square test.

** p-value does not include unknown/missing category.

**Figure 1:**
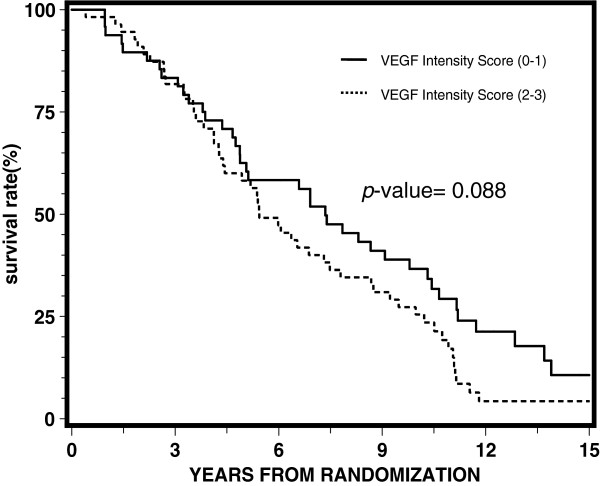
Univariate proportional hazards regression analysis for overall survival.

**Figure 2:**
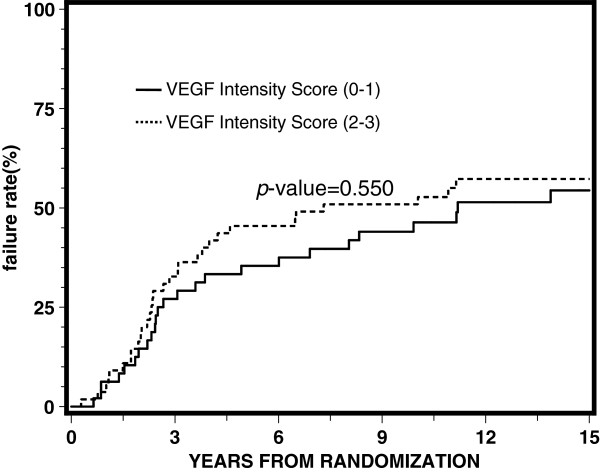
Univariate proportional hazards regression analysis for distant metastasis.

**Figure 3:**
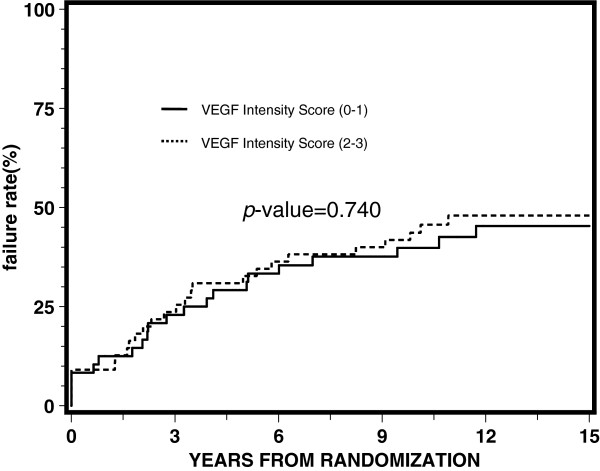
Univariate proportional hazards regression analysis for local progression.

**Figure 4:**
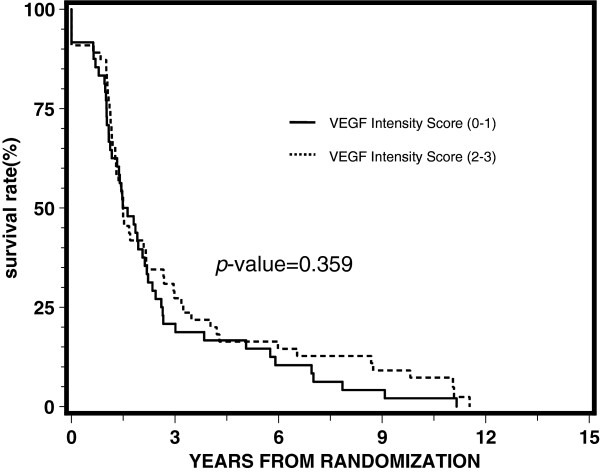
Univariate proportional hazards regression analysis for disease-free survival.

**Figure 5:**
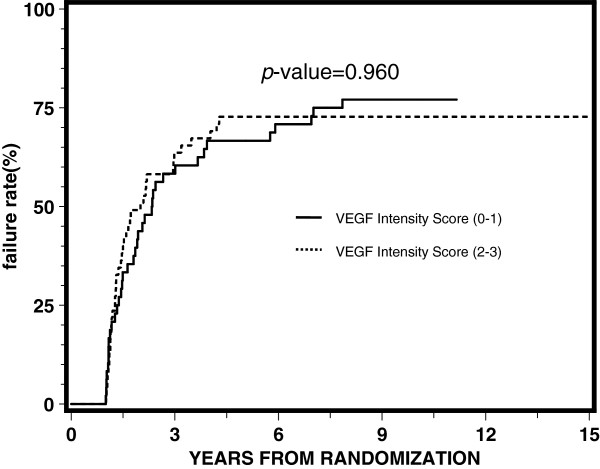
Univariate proportional hazards regression analysis for biochemical failure.

Table 2 reports the multivariate proportional hazards analyses for VEGF expression for each endpoint. As presented in the table, the results, when adjusted for other covariates, also show no significant association of VEGF expression with any of the endpoints. Table 3 reports the results of proportional hazards regression by treatment arm. VEGF expression was not significantly associated with any of the endpoints for each arm, although it appeared to be approaching statistical significance with a p-value of 0.05 for disease-free survival for the STAD + RT arm. Univariate and multivariate analyses demonstrated no statistically significant correlation between the intensity of VEGF expression (0–1) and (2–3) for overall survival, distant metastasis, local progression, disease-free survival, or biochemical failure. Figures 1, 2, 3, 4 and 5 illustrate the graphs for the univariate proportional hazards regression models for each of the above endpoints, portraying the non-statistical significance of VEGF expression for these endpoints studied.

**Table 2:** Multivariate proportional hazards models (n = 103)

**Endpoints**	**Covariate**	**Comparison**	HR^*^	**95% CI**	p-value^**^
**Overall Survival**	VEGF	0-1 vs.	RL		
		2-3	1.314	(0.850, 2.030)	0.219
	Treatment arm	STAD + RT arm vs.	RL		
		RT alone arm	1.268	(0.821, 1.959)	0.285
	Age	< 71 vs.	RL		
		≥ 71	1.708	(1.112, 2.625)	0.015^†^
	Combined Gleason Score	2-6 vs.	RL		
		7-10	1.416	(0.883, 2.270)	0.149
	Clinical Stage	T2 vs.	RL		
		T3	1.026	(0.626, 1.681)	0.918
**Distant Metastasis**	VEGF	0-1 vs.	RL		
		2-3	1.090	(0.629, 1.889)	0.760
	Treatment arm	STAD + RT arm vs.	RL		
		RT alone arm	1.472	(0.861, 2.517)	0.160
	Age	< 71 vs.	RL		
		≥71	1.096	(0.631, 1.903)	0.740
	Combined Gleason Score	2-6 vs.	RL		
		7-10	2.528	(1.358, 4.705)	0.003^†^
	Clinical Stage	T2 vs.	RL		
		T3	0.819	(0.446, 1.504)	0.520
**Local Progression**	VEGF	0-1vs.	RL		
		2-3	1.160	(0.662, 2.030)	0.600
	Treatment arm	STAD + RT arm vs.	RL		
		RT alone arm	1.306	(0.726, 2.349)	0.370
	Age	< 71 vs.	RL		
		≥71	0.873	(0.496, 1.536)	0.640
	Combined Gleason Score	2-6 vs.	RL		
		7-10	0.757	(0.426, 1.344)	0.340
	Clinical Stage	T2 vs.	RL		
		T3	0.657	(0.361, 1.197)	0.170
**Disease-free Survival**	VEGF	0-1vs.	RL		
		2-3	0.859	(0.573, 1.286)	0.461
	Treatment arm	STAD + RT arm vs.	RL		
		RT alone arm	2.102	(1.370, 3.227)	< 0.001^†^
	Age	< 71 vs.	RL		
		≥ 71	0.932	(0.618, 1.407)	0.738
	Combined Gleason Score	2-6 vs.	RL		
		7-10	1.717	(1.048, 2.813)	0.032^†^
	Clinical Stage	T2 vs.	RL		
		T3	0.887	(0.539, 1.458)	0.626
**Biochemical Failure**	VEGF	0-1vs.	RL		
		2-3	1.041	(0.655, 1.652)	0.870
	Treatment arm	STAD + RT arm vs.	RL		
		RT alone arm	1.995	(1.250, 3.186)	0.004^†^
	Age	< 71 vs.	RL		
		≥ 71	0.770	(0.483, 1.229)	0.270
	Combined Gleason Score	2-6 vs.	RL		
		7-10	1.403	(0.785, 2.508)	0.250
	Clinical Stage	T2 vs.	RL		
		T3	0.961	(0.503, 1.837)	0.900

^*^ A hazard ratio (HR) is defined as the ratio of the estimated hazard for those with a variable value 1 to the estimated hazard for those with a variable value 0. A hazard ratio of 1 indicates no difference between two subgroups.

^**^ P-values from Chi-square test using Cox (overall survival and disease-free survival) or Fine & Gray (distant metastasis, local progression, and biochemical failure) Proportional Hazards Model.

^†^ indicates the statistically significant at the significance level of 0.05.

1 patient without Gleason score is not included.

**Table 3:** Multivariate proportional hazards models by treatment arm

**STAD + RT Arm(n = 45)**	**Endpoints**	**Covariate**	**Comparison**	HR^*^	**95% CI**	p-value^**^
	**Overall Survival**	VEGF	0-1 vs.	RL	(0.734, 2.745)	0.299
			2-3	1.419		
		Age	< 71 vs.	RL	(0.608, 2.910)	0.475
			≥ 71	1.330		
		Combined Gleason Score	2-6 vs.	RL	(0.393, 2.581)	0.988
			7-10	1.007		
		Clinical Stage	T2 vs.	RL	(0.213, 1.196)	0.120
			T3	0.505		
	**Distant Metastasis**	VEGF	0-1 vs.	RL	(0.264, 1.326)	0.200
			2-3	0.592		
		Age	< 71 vs.	RL	(0.813, 6.614)	0.120
			≥71	2.318		
		Combined Gleason Score	2-6 vs.	RL	(1.622,61.105)	0.013^†^
			7-10	9.957		
		Clinical Stage	T2 vs.	RL	(0.376, 3.580)	0.800
			T3	1.160		
	**Local Progression**	VEGF	0-1vs.	RL	(0.321, 2.064)	0.660
			2-3	0.814		
		Age	< 71 vs.	RL	**(**0.288**,** 3.515**)**	0.990
			≥71	1.006		
		Combined Gleason Score	2-6 vs.	RL	(0.420, 9.427)	0.390
			7-10	1.990		
		Clinical Stage	T2 vs.	RL	(0.146, 1.755)	0.280
			T3	0.506		
	**Disease-free Survival**	VEGF	0-1vs.	RL	(0.273, 0.995)	0.048^**†**^
			2-3	0.521		
		Age	< 71 vs.	RL	(0.807, 3.601)	0.162
			≥ 71	1.705		
		Combined Gleason Score	2-6 vs.	RL	(0.792, 4.567)	0.150
			7-10	1.902		
		Clinical Stage	T2 vs.	RL	(0.403, 2.054)	0.052
			T3	0.910		
	**Biochemical Failure**	VEGF	0-1vs.	RL	(0.251, 1.170)	0.120
			2-3	0.542		
		Age	< 71 vs.	RL	(0.805, 3.834)	0.160
			≥ 71	1.757		
		Combined Gleason Score	2-6 vs.	RL	(0.783, 14.343)	0.100
			7-10	3.352		
		Clinical Stage	T2 vs.	RL	(0.425, 3.151)	0.780
			T3	1.157		
**RT Alone Arm(n = 58)**	Endpoints	Covariate	Comparison	HR^*^	95% CI	p-value^**^
	**Overall Survival**	VEGF	0-1 vs.	RL	(0.622, 2.028)	0.699
			2-3	1.123		
		Age	< 71 vs.	RL	(0.890, 2.800)	0.118
			≥ 71	1.579		
		Combined Gleason Score	2-6 vs.	RL	(0.839, 2.566)	0.178
			7-10	1.468		
		Clinical Stage	T2 vs.	RL	(0.760, 2.917)	0.246
			T3	1.489		
	**Distant Metastasis**	VEGF	0-1 vs.	RL	(0.777, 3.319)	0.200
			2-3	1.606		
		Age	< 71 vs.	RL	(0.422, 1.706)	0.640
			≥71	0.848		
		Combined Gleason Score	2-6 vs.	RL	(0.992, 3.825)	0.053
			7-10	1.947		
		Clinical Stage	T2 vs.	RL	(0.357, 1.789)	0.590
			T3	0.800		
	**Local Progression**	VEGF	0-1vs.	RL	(0.670, 2.669)	0.410
			2-3	1.337		
		Age	< 71 vs.	RL	(0.391, 1.643)	0.540
			≥71	0.801		
		Combined Gleason Score	2-6 vs.	RL	(0.259, 1.067)	0.075
			7-10	0.525		
		Clinical Stage	T2 vs.	RL	(0.415, 1.971)	0.800
			T3	0.905		
	**Disease-free Survival**	VEGF	0-1vs.	RL	(0.779, 2.371)	0.279
			2-3	1.359		
		Age	< 71 vs.	RL	(0.305, 0.979)	0.042^†^
			≥ 71	0.547		
		Combined Gleason Score	2-6 vs.	RL	(1.021, 3.537)	0.043^†^
			7-10	1.900		
		Clinical Stage	T2 vs.	RL	(0.473, 1.913)	0.889
			T3	0.952		
	**Biochemical Failure**	VEGF	0-1vs.	RL	(0.815, 2.562)	0.210
			2-3	1.446		
		Age	< 71 vs.	RL	(0.315, 1.104)	0.056
			≥ 71	0.565		
		Combined Gleason Score	2-6 vs.	RL	(0.655, 2.356)	0.510
			7-10	1.242		
		Clinical Stage	T2 vs.	RL	(0.474, 2.459)	0.850
			T3	1.080		

^*^ A hazard ratio (HR) is defined as the ratio of the estimated hazard for those with a variable value 1 to the estimated hazard for those with a variable value 0. A hazard ratio of 1 indicates no difference between two subgroups.

^**^ P-values from Chi-square test using Cox (overall survival and disease-free survival) or Fine & Gray (distant metastasis, local progression, and biochemical failure) Proportional Hazards Model.

^†^ indicates the statistically significant at the significance level of 0.05.

1 patient without Gleason score is not included.

## Discussion

The present study represents one of the largest sample bases with long-term follow-up reviewed for VEGF expression in human prostate carcinoma patients, providing much needed incremental progress in the field of understanding angiogenic factors and modern biomarkers for prostate cancer prognosis, which to date has only been investigated in a limited number of studies. Although VEGF expression has been well-studied in many other malignancies, much controversy exists in the literature for VEGF expression and its prognostic and predictive value for prostate carcinoma [18-28,31-34]. The need for further understanding in this area of prostate cancer research is thus urgently needed.

The natural history of prostate carcinoma is one with a wide spectrum, ranging from being relatively indolent where a patient may have a life expectancy similar to the general population, to being aggressive with rapid development of metastases and ultimately death [35,36]. Currently, with conventional strategies, clinicians have only a moderate ability to estimate prognosis in patients with newly diagnosed prostate cancer, and subsequently there is uncertainty regarding the optimal patient management. These conventional prognostic indicators include clinical tumor stage, Gleason score, and pre-treatment serum prostate specific antigen (PSA) levels [37]. The development of novel prognostic and predictive biomarkers is thus crucial to identify patients who may benefit from further specific therapy. A prognostic biomarker furnishes information regarding the patient’s overall cancer outcome, irrespective of therapy, while a predictive biomarker may predict response to a particular treatment [38].

Although commonly used tests for women with newly diagnosed breast cancer include estrogen and progesterone receptors and HER2 status, which have both prognostic and predictive value, comparable molecular markers are not available for men with a diagnosis of prostate cancer. Through these molecular markers, appropriate targeted therapy may be selected for patients at a higher risk of cancer progression who will benefit from treatment and avoiding the side effects of therapy from those who will not benefit. Thus, the search for a biomarker of similar utility in prostate cancer is of great importance. A recent review of classical and novel biomarkers as prognostic factors for prostate cancer has highlighted the poor quality and heterogeneity of studies to date, with generally no conclusive results thus far [39].

VEGF is a potent angiogenic factor involved in tumor angiogenesis and represents a potential therapeutic target. Published data regarding VEGF expression in prostate carcinoma has been conflicting. Previous studies in the literature have reported markedly varied VEGF expression in prostate carcinoma, ranging generally from 40% to 100% [18-28]. There are limited, even fewer, studies on the prognostic and predictive value of VEGF expression in prostate cancer, also with controversial results [31-34].

In the present study, which is among the largest studies of VEGF expression in prostate cancer with a long-term follow-up of 12.2 years, we explored the potential value of VEGF as a prognostic and predictive biomarker among men with locally advanced prostate cancer enrolled on RTOG 8610. In this study, we found no statistically significant correlation between the intensity of VEGF expression (0–1) and (2–3) for overall survival, distant metastasis, local progression, disease-free survival, or biochemical failure. The results remain non-significant when adjusted for other covariates. There is also no statistically significant predictive value of VEGF demonstrated comparing the treatment arms.

These results are consistent with recent works such as that by Concato *et al*. [31] who investigated molecular markers of cell cycle regulation and angiogenesis and death from prostate cancer. The authors found that there is immunohistochemical evidence of association of bcl-2, p53, or high microvessel density in prostate cancer biopsy specimens at diagnosis and an increased long-term risk of death from prostate cancer. However, there was no statistically significant association with VEGF. A study by Strohmeyer *et al*. [40] also found the importance of microvessel density. In particular, they noted that a high microvessel density was a negative prognostic factor for tumor progression and had a predictive value higher than the classical characteristics of clinical stage, grade, and PSA in prostate cancer after radical prostatectomy. On the contrary, previous reports in the literature by Peyromaure *et al.*[32] found that VEGF expression in prostate cancer tissue was associated with the risk of cancer progression after radical prostatectomy. Furthermore, Shariat and colleagues [33] reported that preoperative plasma VEGF was independently associated with metastases to lymph nodes and biochemical progression after radical prostatectomy, and Vergis and colleagues [34] found that increased expression of VEGF and HIF-1alpha were noted in patients at high risk of biochemical failure. It is also interesting to note that Mori and colleagues [41] have recently suggested prognostic value for VEGF-A and VEGF-C expression levels in that higher VEGF-A expression was associated with improved overall survival and high VEGF-C expression was associated with decreased risk of developing clinical recurrence. They examined gene expression levels using quantitative real-time PCR. It also differed from our study in that it represented a post prostatectomy setting while our study population consisted of patients treated with primary radiation therapy. The findings of increased VEGF levels in the castrate disease population may suggest that tumor cells acquire new alterations that enable them to survive in the castrated state (adaptation), or represent the outgrowth of rare, pre-existing cells capable of surviving hormonal therapy (selection). The patient population of RTOG 8610 represents a locally-advanced disease population with bulky tumors. The role of elevated VEGF levels in the castrate resistant population may reflect an adaption response, as these patients are on long-term (life) androgen deprivation therapy. The paper by Mori and colleagues [41] evaluated short-term androgen deprivation therapy in a preoperative vs. post-operative setting. A possible difference in the findings may be a function of duration of androgen deprivation therapy in the respective patient populations.

There are a number of confounding factors and limitations that may explain the conflicting results in the literature of VEGF expression studies [18-28,31-34]. Firstly, the majority of these studies consist of retrospective analyses of very small sample sizes with short follow-up. Ideally, these studies should have long-term follow-up, such as our present study, to examine endpoints such as overall survival rather than biochemical failure. Secondly, measurement of VEGF levels is often problematic, with issues involving tissue availability, sample collection, tissue processing, and storage techniques, all of which could alter results [42]. In fact, in a study of renal cell carcinoma, Jacobsen and colleagues [43] noted that increased storage time resulted in decreased VEGF expression in the membranes of tumor cells from paraffin-embedded tissue samples. Christensen and colleagues [42] suggest that, for reliable and consistent results, the ideal conditions for sample collection and preparation should be identified in the study design phase. Then each aspect of sample collection, processing, and storage should be clearly specified in the standard operating procedure document of the study. Thirdly, the level of sensitivity of commonly used assays may be too low to detect meaningful changes in VEGF expression, as even small changes in tumor VEGF expression may be clinically significant depending on the level of dependence of the tumor on VEGF signaling [44]. Furthermore, the lack of significance as found in this study may be due to the tissue sample size and homogeneity of the patient group. Lastly, there is no consensus in the literature of a “gold standard” VEGF detection assay, and there is a lack of a predefined, accepted, clinically meaningful “cut-off” point for VEGF expression assay measurements. The authors acknowledge that there are other quantitative methods of evaluating VEGF such as examining mRNA or gene expression levels; however, at the time this analysis was performed, the IHC method was the RTOG “standard” at that time and continues to be widely used. Examining the VEGF receptor may further provide informative information in future studies.

In conclusion, the results of much research in VEGF expression in human prostate cancer to date are conflicting. Most studies are only exploratory or hypothesis generating, with small patient numbers. There are even fewer studies on the prognostic or predictive value of VEGF in prostate cancer. Trials are often fraught with inherent challenges as described above. Euphoria is now somewhat tempered because often the initially reported promising results are not reproducible.

In our present study, we found no statistically significant prognostic or predictive value of VEGF expression for locally advanced prostate cancer. This study is among the larger studies of VEGF expression in prostate cancer, and we urge the research community to avoid the misrepresentation of the literature with a lack of publication of even well-designed large negative studies, a publication bias against negative trials, as the current literature in this area appears to be predominated by only small exploratory positive trials, with a lack of subsequent confirmation with larger, longer prospectively designed trials. Thus, to date, the usefulness of VEGF as a prognostic and predictive factor in prostate cancer remains to be clarified. In this study, we had the opportunity to evaluate VEGF levels in the well-characterized RTOG 8610 patient population. There have been a number of other biomarker studies done in this patient population already, and this study complements what is known for this patient population [45-51]. However, with the limitations presented, we acknowledge that this secondary analysis of RTOG 8610 will not serve to make the definitive statement regarding whether VEGF is a useful biomarker or not, but reporting on this well-characterized patient population with long-term follow-up and numerous other biomarker publications arising from this population is in our opinion a significant contribution to the current heterogeneous VEGF literature [18-28,31-34].

There is an urgent need to establish multidisciplinary initiatives for coordinating further research in the area of human prostate cancer biomarkers, and ultimately strive towards improving the treatment of prostate cancer patients through better targeted therapy.

## Competing interests

The authors declare that they have no competing interests.

## Authors’ contributions

RTOG believes strongly in recognizing investigators who significantly contributed to the scientific development of the study/project, the data analysis, and manuscript writing and review, as well as those who provide scientific data (patient accrual, clinical data, and biological material submission). The authorship line is determined by the RTOG Publications Committee in accordance with the RTOG Publications Guidelines, which can be found at http://www.rtog.org. All authors read and approved the final manuscript.
